# The Efficacy of Erector Spinae Plane Block for Patients Undergoing Percutaneous Nephrolithotomy

**DOI:** 10.4274/TJAR.2022.22981

**Published:** 2023-06-16

**Authors:** Mehmet Uğur Bilgin, Zeki Tuncel Tekgül, Tansu Değirmenci

**Affiliations:** 1 Clinic of Anaesthesiology and Reanimation, University of Health Sciences Turkey, İzmir Bozyaka Training and Research Hospital, İzmir, Turkey; 2 Clinic of Urology, University of Health Sciences Turkey, İzmir Bozyaka Training and Research Hospital, İzmir, Turkey

**Keywords:** Erector spinae plane block, nerve block, opioid, pain management, percutaneous nephrolithotomy, regional anaesthesia

## Abstract

**Objective:**

Percutaneous nephrolithotomy (PCNL) is accompanied by somatic and visceral pain intraoperatively and postoperatively. However, pain management strategies lack a decisive consensus. Erector spinae plane block (ESPB) is a novel paraspinal fascial block that can be used in PCNL patients, and we aimed to investigate whether ESPB will reduce intraoperative and postoperative opioid consumption and postoperative pain scores in PCNL patients.

**Methods:**

The study was randomized, controlled, and open-label. Two groups were formed as the control group (GCont) and block group (Gblock), and patients received total intravenous anaesthesia. GBlock received an ESPB catheter in addition in the prone position. Intraoperative parameters and infusion doses, postoperative rescue analgesic doses, and pain scores were recorded. The primary endpoint was intraoperative analgesic consumption, and the secondary endpoints were postoperative pain scores and analgesic consumption.

**Results:**

Sixty-four patients were analyzed. Remifentanil consumption of GCont was found to be significantly higher (GBlock: 0.0865 ± 0.030 vs GCont: 0.1398 ± 0.034, μg kg^-1^ min^-1^, *P* < 0.001). The control group reported higher pain scores between the 30^th^ min and 24^th^ hours and needed more analgesics between the 1^st^ and 6^th^ hours postoperatively. GBlock received local anaesthetics via ESPB catheter before nephrostomy tube removal and fewer patients needed analgesics [5 patients (15.6%) vs. 28 patients (87.5%), *P* < 0.001]. GCont consumed more tramadol postoperatively (262.5 mg vs. 75 mg, *P* < 0.001).

**Conclusion:**

We found that ESPB reduced intraoperative opioid consumption. It also reduced the need for rescue analgesia and postoperative pain scores during nephrostomy tube removal. We concluded that the ESPB catheter may effectively be used in analgesia management during and after PCNL operations.

**Main Points**

• Our results showed that erector spinae plane block (ESPB) reduces intraoperative and postoperative opioid consumption and postoperative pain scores.

• In addition, providing effective analgesia during nephrostomy catheter tube removal proves that ESP improves both visceral and somatic pain.

• Thus, we believe that the ESPB is a reliable analgesia option for percutaneous nephrolithotomy patients.

## Introduction

Percutaneous nephrolithotomy (PCNL) may lead to severe postoperative pain. Acute pain may originate from the skin, muscles, renal capsule, renal parenchyma, and ureter. Nephrostomy tube removal and ambulation also cause visceral and somatic stimuli.^1^ Visceral pain originating from the kidneys and ureters is transmitted via T10-L1 and T10-L2.^2^ The cutaneous innervation of the incision site mainly originates from T10-T11 (T8-T12). Systemic analgesics, regional anaesthesia, small diameter nephrostomy tube or tubeless surgery, and local analgesic infiltration techniques have been tried to improve postoperative pain management in PCNL patients.^3,4,5,6,7,8^

Erector spinae plane block (ESPB) was described by Forero et al.^9^ in 2016 as an analgesic method for thoracic neuropathic pain. It provides unilateral analgesia of the anterior and posterior chest wall by craniocaudal spreading of the local anaesthetics (LA) applied below the fascia of the erector spinae muscle group (ESMG). By changing the injection site caudally, sensation in the abdomen and lumbar region can be blocked.^10^

We hypothesized that ESPB would be a safe and effective analgesia option for PCNL operations and designed our study to evaluate the effectiveness of ESPB for both intraoperative and postoperative analgesia.

We designated our primary goal as comparing the groups for intraoperative opioid consumption. Our secondary goals included comparing the pain scores and opioid consumption of the groups during nephrostomy tube removal, ambulation, and at certain postoperative hours.

## Methods

The study was conducted as prospective, randomized, controlled, and open-label in a tertiary referral hospital. We followed the CONSORT 2010 guidelines and adhered to the Declaration of Helsinki. Ethics Committee for Clinical Studies of University of Health Sciences University Turkey, İzmir Bozyaka Training and Research Hospital approved the study with approval no: 5, date: 04.07.2018. Study design was also registered and approved in the ClinicalTrials.gov with the number NCT03652103. The eligible patients were informed about the study during the preoperative evaluation and their written consents were obtained.

A preliminary study was conducted for statistical power analysis based on the average intraoperative opioid consumption. The effect size was calculated as 0.936, with 95% statistical power and 5% type 1 error margin, and the sample size as at least 31 patients per group and 62 in total. 70 patients were enrolled, with the expectation of 62 patients in the end, estimating a drop-off rate of around 10-15%. The patients were assigned to either the Erector Spinae Plane Block Group (GBlock) or the Control Group (GCont) based on computer-generated randomisation.

**Figure 1:**
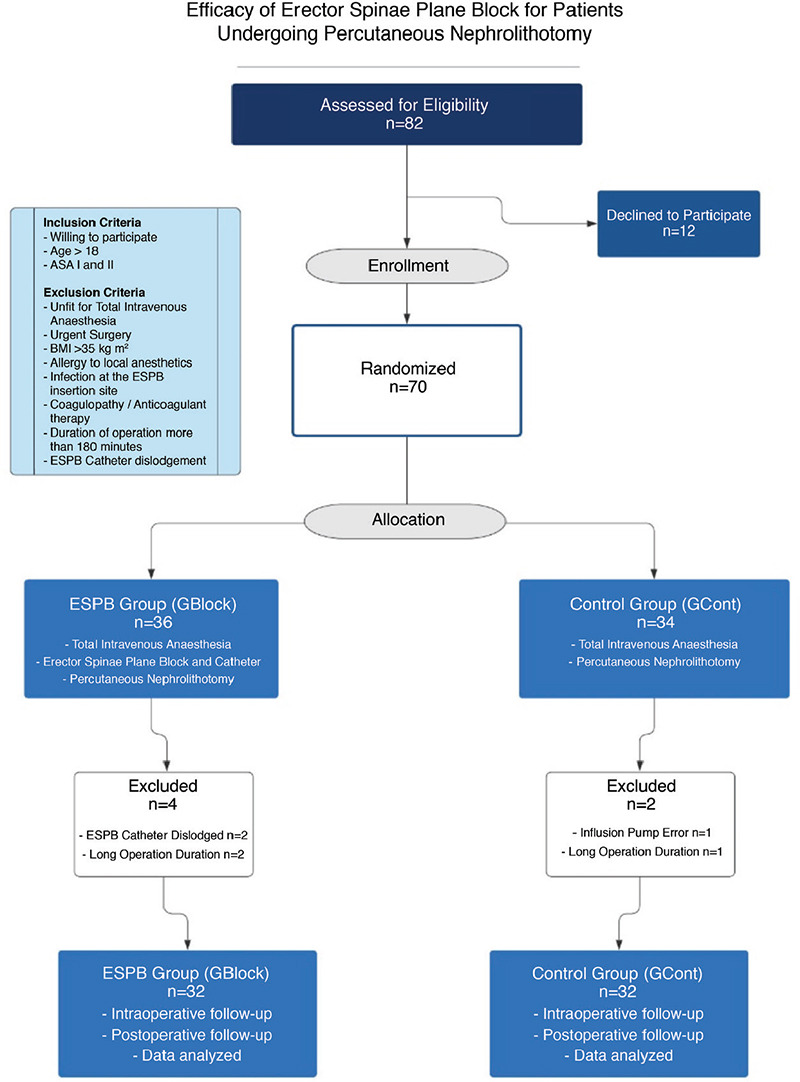
Flow chart. ESPB, erector spinae plane block; BIS, bispectral index; GBlock, block group; GCont, control group.

Patient admission started on September 05, 2018 and ended on March 10, 2019. Patients older than 18 years with American Society of Anesthesiologists Physical Status I or II and 2-3 cm renal stones were included. Preoperative evaluation included coagulation profile, serum creatinine levels, urinalysis, urine culture, and computed tomography scan for urinary tract. Inclusion and exclusion criteria are further explained in the flowchart. After the excluded patients, the study was completed with 64 patients (Figure 1).

In the operating room, patients were monitored with electrocardiogram, blood pressure, pulse oximeter, and bispectral index (BIS). 4-electrode BIS Quatro^®^ (Covidien IIc, USA) sensors were used for BIS monitoring. Induction was carried out with lidocaine 1.5 mg kg^-1^, propofol 2 mg kg^-1^, rocuronium 0.5 mg kg^-1^ and remifentanil 1 µg kg^-1^ IV. Total intravenous anaesthesia (TIVA) was commenced with propofol at a rate of 100 µg kg^-1^ min^-1^ and remifentanil at a rate of 0.07 µg kg^-1^ min^-1^ IV. For preventive analgesia, paracetamol 10 mg kg^-1^ IV was administered to all patients after the induction. The anaesthesia management of the patients was carried out by the primer anaesthesiologist of the room who was informed about the study. Hemodynamic and BIS parameters were recorded every five minutes, but the comparison of the groups for hemodynamic and BIS changes was limited to the shortest operation time to include all patients. The propofol infusion dose was titrated so that the BIS value of the patients was between 40 and 60^11^; the remifentanil infusion dose was titrated so that the heart rate and mean arterial pressure values ​​remained within ± 20% of the patient’s baseline.

After intubation, patients were positioned in the lithotomy position for ureteroscopy (URS). A ureteral catheter was placed. After retrograde pyelography was performed, patients were placed in the prone position for PCNL. All operations were performed by the same primary attending surgeon, who is also the co-author. Patients in the block group (GBlock) received the ESPB in the prone position after URS. ESPB catheter insertion sites were tailored to the location of the stone, thus surgical incision area, between T7-T10 vertebra levels. With these adjustments, we aimed to align the tip of the catheter with the mid-level of the kidney nerve roots and incision site in the medulla to standardize the LA injection site in all cases.

**Figure 2:**
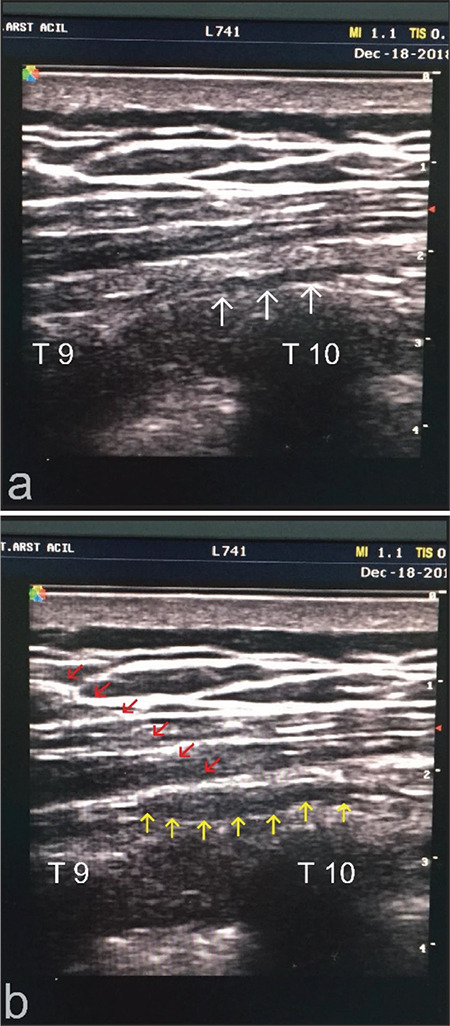
Ultrasound image of ESPB. a. Before the application of local anaesthetic. b. After the application of local anaesthetic through the catheter. T9: Costotransverse joint of T9 vertebrae, T10: Costotransverse joint of T10 vertebrae, white arrows: the lower fascia of erector spinae muscle group, red arrows: shadow of the erector spinae block catheter, yellow arrows: local anaesthetic filled area. ESPB, erector spinae plane block.

Following the marking of the thoracic vertebrae levels with a marker pen, the skin area was prepared with Povidone-iodine solution. The intervention was initiated when the transverse processes and the costotransverse joints on the relevant level were distinguished using a linear ultrasound probe. The catheter needle was inserted at a 30° angle to the skin in an in-plane and craniocaudal fashion. The tip of the needle was passed through the lower fascia of the ESMG and halted above the costotransverse joint. 5 mL normal saline (0.9%) was injected to confirm the localization of the needle tip and to aid catheter advance by hydrodissection. The catheter was advanced 2 to 4 cm inside to reach the designated level and reduce the risk of dislocation. Proper placement was confirmed with ultrasonography and then 20 mL of 0.25% bupivacaine solution was administered through the catheter for intraoperative and postoperative analgesia (Figure 2).

When the surgical procedure was completed, TIVA was stopped and sugammadex 4 mg kg^-1^ IV was administered to reverse the neuromuscular blockade. Patients were questioned for pain (with the Numerics Rating Scale - NRS) before leaving the operating room. This was recorded as 0^th^-minute-NRS, and, if necessary, the rescue analgesics was administered. Rescue analgesia was planned to be tramadol 1 mg kg^-1^ IV (400 mg day^-1^ maximum) when patients’ pain scores (NRS) were 3 or above. The patients were then transferred to the post-anaesthesia recovery unit (PACU).

Paracetamol 10 mg kg^-1^ IV was prescribed to every patient in the study every 8 h for postoperative analgesia. Patients’ questionnaires for pain scores were scheduled at the end of the operation at the 30^th^ and 60^th^ minutes in the PACU and at the 2^nd^, 6^th^, 12^th^, 24^th^, and 48^th^ hours in the wards. In these questionnaires, rescue analgesic administrations were also recorded.

Patients were encouraged to ambulate on postoperative day (POD) 0, approximately 6 h after the operation. Patients in the GBlock group were administered 20 mL 0.25% bupivacaine solution via the ESPB catheter 30 min before ambulation. GCont patients did not receive any medications before ambulation with the motivation not to interfere with true analgesic consumption and to administer the same postoperative systemic analgesia protocol for both groups. Pain scores of the patients during ambulation were recorded as NRS.

On POD 1, GBlock received LA dose (20 mL 0.25% bupivacaine) twice at 12-h intervals. Both groups received rescue analgesics when their NRS scores exceeded 3 out of 10.

Nephrostomy tubes were removed at POD 2. Patients in the GBlock group received the same LA dose through the ESPB catheter 30 min in advance. For the same reasons as in ambulation, GCont did not receive any medication before tube removal. NRS scores of the patients during the removal of the nephrostomy tube were recorded.

ESPB- or LAs related complications (LA systemic toxicity, insertion site infection, muscle weakness, and pneumothorax) were monitored both intraoperatively and postoperatively.

### Statistical Analysis

IBM Statistical Package for Social Sciences version 24 was used for statistical calculations. The compliance of the data to the normal distribution was determined by the single sample Kolmogorov-Smirnov test. Normally distributed quantitative data were compared with the independent sample *t*-test, and quantitative data that did not follow the normal distribution were compared with the Mann-Whitney U test. Chi-square test was used to compare qualitative data. Parametric test results were reported as mean and standard deviation and nonparametric test results as number and percentage or median and interquartile range.

The significance level was determined as *P* < 0.05 at the 95% confidence interval.

## Results

The groups were similar in terms of age, height, weight and education level (Table 1).

The remifentanil consumption of the GCont was found to be significantly higher (*P *< 0.001). The groups were comparable in terms of other intraoperative data (Table 2).

GCont reported statistically significantly higher pain scores between the 30^th^ minute and the 24^th^ hour postoperatively (*P *< 0.001, *P*=0.002, *P *< 0.001, *P *< 0.001, *P *< 0.001, and *P*=0.015, respectively) (Table 3). However, rescue analgesic doses were found to be different only at the 60^th^ minute, 2^nd^ hour, and 6^th^ hour (*P*=0.003, *P*=0.002, *P*=0.002, respectively). Comparison of pain scores and the number of patients who needed rescue analgesics during ambulation (*P *< 0.001 and *P *< 0.001, respectively) and removal of the nephrostomy tube (*P *< 0.001 and *P *< 0.001, respectively) showed significant differences between the groups. The median postoperative rescue analgesic dose was 75 mg [100, IQR] for GBlock and 262.5 mg [113, IQR] for GCont (*P *< 0.001) (Table 4).

**Figure 3:**
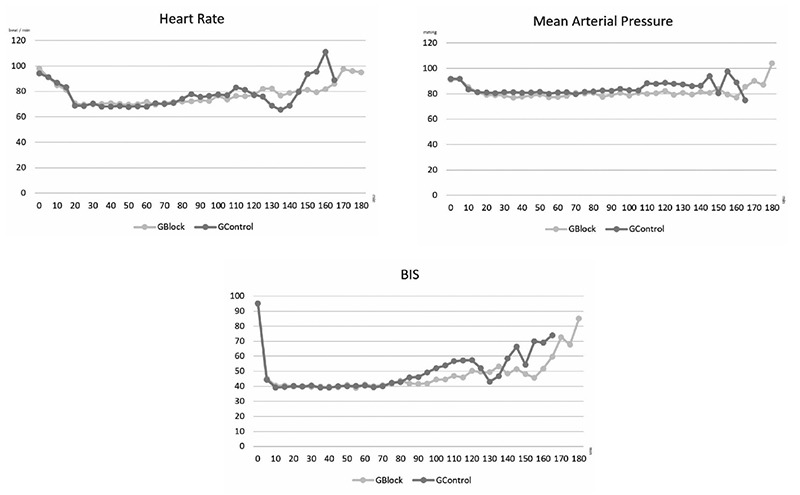
Intraoperative hemodynamic and BIS measurements. BIS, bispectral index; GBlock, block group; GCont, control group.

Heart rate, mean arterial pressure, and BIS values were measured every 5 min during the surgery, and groups were comparable regarding changes in these parameters during the operation (Figure 3).

We did not detect any side effects or complications related to ESPB or LAs.

## Discussion

Efforts to provide a better understanding of the analgesic action mechanism of ESPB are on the rise. Even though it is not yet decisive, common opinion for the action of the mechanism is the LA effect at the ventral and dorsal rami of the spinal nerves. LA applied beneath the lowest fascia of ESMG spreads both craniocaudally and through the intertransverse space. This propagation provides analgesia and sympathetic blockage in a large area by interrupting the anterior, posterior, and communicating rami.^12,13^

ESPB was defined as a single shot injection block, but catheter placement for postoperative use has also been shown for various cases.^14^ Single-shot ESPB studies show a pain ameliorate effect lasting up to 6-12 hours.^15,16^ Even though there are case reports using LA infusions for ESPB catheters, bolus doses can be chosen, as in our study, due to the adequate duration and convenience of use. Oezel et al.^17^ also marked that single-shot or catheter placement for ESPB has no superiority over one another.

ESPB is mentioned in many studies as a successful postoperative analgesia method. A study of 66 patients investigated whether ESPB provides benefits for pain in laparoscopic cholecystectomy. Authors marked that the postoperative NRS scores and opioid consumption in the ESPB group were lower.^18^ Mostafa et al.^19^ conducted a study investigating pain in laparoscopic bariatric surgery with a similar insertion site for ESPB as our study. They compared bilateral ESPB with a bilateral sham block at the T7 level and reported that ESPB provides “satisfactory postoperative analgesia with decreased analgesic consumption without significant difference in postoperative pulmonary functions.” On the other hand, data regarding the intraoperative use of ESPB is in short supply and we aimed to fill this gap with our study.

ESPB has recently been shown to be effective for postoperative analgesia after PCNL operations.^20,21^ To our knowledge we conducted the first randomized and controlled study to investigate the effectiveness of ESPB intraoperatively in PCNL patients. Our hypothesis regarding the use of ESPBs in PCNL operations is consistent with our results. Intraoperative TIVA infusion doses combined with similar BIS and hemodynamic changes in both groups, exhibited a valuable outcome. We found a statistically significant difference in remifentanil infusion doses between the groups (GBlock: 0.0865 µg kg^-1^ min^-1^ vs. GCont: 0.1398 µg kg^-1^ min^-1^, *P* < 0.001). Painful stimuli during surgery cause stress response, which leads to hemodynamic changes (e.g. tachycardia and hypertension), increased catabolism, hyperglycemia, hypercoagulability, salt and water retention (hence edema), etc.^22^ Lower doses of remifentanil infusion with similar hemodynamic readings between the groups clearly indicate that ESPB performed before surgery can reduce pain during surgery. Considering the unfavorable effects of surgical stress caused by noxious stimuli,^23^ we can argue that the benefit of ESPB in PCNL patients may reach beyond just analgesia. This result successfully concludes our primary endpoint.

Our secondary outcomes are also promising. We detected that ESPB reduced postoperative pain in PCNL patients. GBlock described lower pain scores from the 30^th^ minute to 24^th^ hour postoperatively compared with GCont. But rescue analgesics were administered to GCont mostly from 60^th^ minute to 12^th^ hour. Both groups demanded very little opioids after 24^th^ hour. We think that the difference in opioid use in the first 12 hour and the decrease of this difference in the following hour may point out that POD 0 is more significant for pain management in PCNL patients. In terms of postoperative total opioid consumption, patients in the block group received 75 [100] mg (median [IQR]) and the patients in the control group received 262.6 [113] mg (median [IQR]) tramadol IV (*P* < 0.001). These results reveal the success of ESPB in reducing postoperative pain and analgesic consumption in PCNL.

Nephrostomy tubes increase the acute pain after PCNL and patients mainly experience distress during the tube removal.^6,24^ These tubes are in direct contact with the skin, kidney capsule, and kidney parenchyma and create noxious stimulation in these tissues during withdrawal. To alleviate nephrostomy tube removal pain, we would need to prevent both visceral and somatic stimuli at the relevant levels. The fact that we found a statistically and clinically significant difference in pain scores during removal of the nephrostomy tube stands out as an important finding in terms of the coverage of the ESPB. As for rescue analgesia, 28 (87.5%) patients in the control group and only 5 (15.6%) in the block group demanded rescue analgesia during nephrostomy tube removal. This result, along with the reduction of intraoperative opioid doses, can be interpreted as the two most striking clinical outcomes of this study.

Reducing pain during ambulation and thus facilitation of ambulation helps to avoid many postoperative complications such as ileus, edema, venous thromboembolism, et cetera.^25,26^ We determined that the average NRS of GCont patients was 4 and GBlock patients was 2 during ambulation. Thirty-one patients (96.9%) in the control group requested rescue analgesic after ambulation. In the block group, the number was 19 (59.4%) (*P* < 0.001). Although a statistically significant difference was found, the fact that there was no clear difference as with nephrostomy tube removal suggests that visceral stimuli with a wider range of effects are at the forefront during ambulation. ESPB increased patient comfort during ambulation but could not provide sufficient analgesia alone.

Since its first publication, many studies have reported that ESPB has few or no complications and most of the known complications are due to LAs.^27,28^ Consistent with these results, we detected no side effects or complications in any patient.

Our study has two main limitations: open-label design and the lack of a sham block group.

Open-label designs may put the integrity of studies into question. However, for our primary outcome, intraoperative dose titrations were strictly designated according to BIS and hemodynamic monitoring before the study, and we believe that the lack of physicians’ blindness did not result in an increase in bias in intraoperative data.

The lack of a sham block group was intentionally decided to avoid unnecessary invasive intervention, considering that patients receive general anaesthesia for the procedure. We accept the criticism, particularly for postoperative pain follow-ups where evaluators (and patients) were aware of the questioned patient group.

The strength of our study comes from the strict, objective measurements of intraoperative data. Pain assessments are mainly subjective and may be affected by the perception of assessment scales, environment, and patient characteristics Postoperative pain assessments are valuable and may even be irreplaceable for pain investigation studies, considering that pain itself is subjective by definition.^29^ However, we believe our result on intraoperative remifentanil consumption with hemodynamic consistency is a more reliable outcome for the success of ESPB compared with subjective postoperative pain assessments.

## Conclusion

We concluded that with decreased opioid consumption both intraoperatively and postoperatively, analgesic efficiency, low profile of complications and side effects, and ease of application, the erector spinae plane block stands out as a useful technique for pain management in patients who will undergo PCNL operation.

## Figures and Tables

**Table 1:**
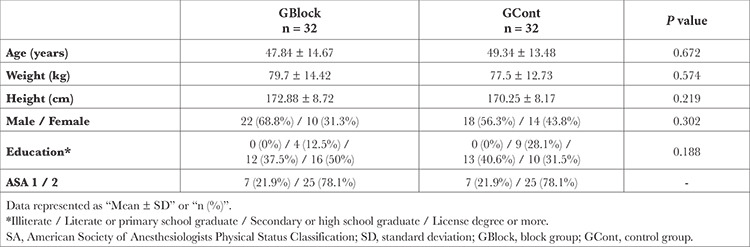
Demographics

**Table 2:**
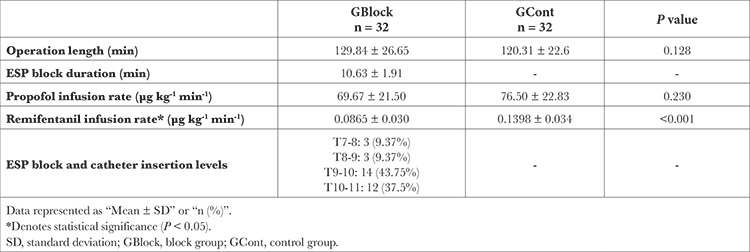
Intraoperative Parameters

**Table 3:**
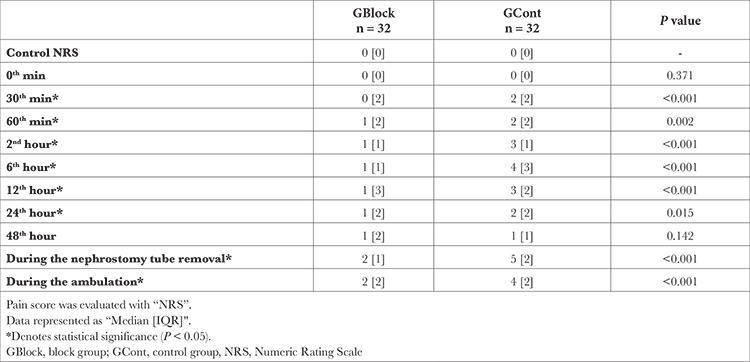
Postoperative Pain Scores

**Table 4:**
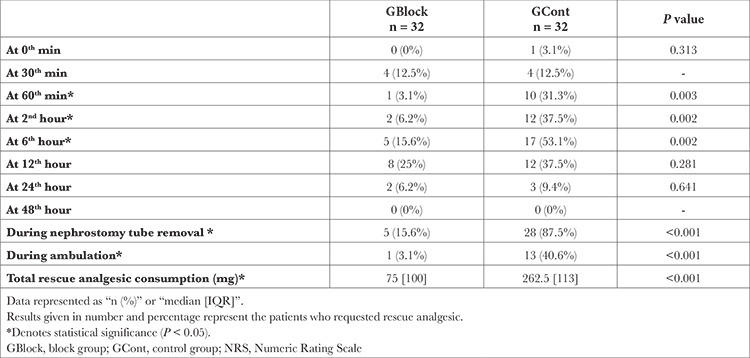
Rescue Analgesic Consumption
